# Robust icephobic coating based on the spiky fluorinated Al_2_O_3_ particles

**DOI:** 10.1038/s41598-021-84283-w

**Published:** 2021-03-08

**Authors:** Anton Starostin, Vladimir Strelnikov, Viktor Valtsifer, Irina Lebedeva, Irina Legchenkova, Edward Bormashenko

**Affiliations:** 1 Institute of Technical Chemistry, UB RAS, Academician Korolev St., 3, Perm, 614013 Russian Federation; 2 Engineering Faculty, Chemical Engineering Department, Ariel University, POB 3, 407000 Ariel, Israel

**Keywords:** Synthesis and processing, Physics, Chemical physics

## Abstract

Omniphobic and icephobic twin-scale surfaces based on the “urchin”-like fluorinated Al_2_O_3_ particles are presented. Combined effect of hierarchical topography and fluorination supplied to the surfaces omniphobic and icephobic properties. The study of the stability of the Cassie wetting state is reported. High apparent contact angles were accompanied with the low contact angle hysteresis and high stability of the Cassie air trapping wetting state. Time delay of the ice crystallization as high as $88\pm 5$ min was established when compared to the ice formation on flat aluminum and non-fluorinated “urchin”-like surfaces. Crystallized water droplets formed on the reported nano-structured surfaces were easily blown out by the air jet with the velocity of $v=3.0\pm 1.0$ m/s, (which is markedly lower than that common for exploitation of aircrafts and turbines). Heated “urchin”-like surfaces completely restored their omniphobic and icephobic surfaces after thawing. Qualitative analysis of water freezing is supplied.

## Introduction

Icing is a widespread phenomenon in subzero climates where water and/or moisture are present and it crucially influences the quality of life and industry including transportation systems, power transmission, infrastructures and energy supply systems^1^. The problem of creating of ice-phobic surfaces attracted the attention of researchers in the last decade^1–9^. The notion of “icephobicity” is ambiguous^1–3^. There exist are at least three very different approaches to the interpretation of icephobicity, as discussed in Ref.^3^. First, icephobicity implies relatively low energy of adhesion (as estimated with the Young–Dupre equation^10–12^) between ice crystals and a supporting surface. It was demonstrated in Ref.^11^ that the ice adhesion strength correlates with the work of adhesion required to remove a liquid water drop from each smooth surface. The authors of Ref.^11^ studied flat strongly hydrophobic surfaces and concluded that reduction in ice adhesion strength will require developments of specially textured surfaces, as no known materials exhibit receding water contact angles on smooth surfaces that are above those reported in Ref.^11^. And this is an approach suggested in the present paper in which icephobic properties of the textured urchin-like surfaces are addressed. The reasonable question is: how the ice adhesion strength should be quantified? The most widespread approach is based on the measurement of the critical shear stress necessary for de-icing^13–16^, although the normal stress is used as well^3^.

The alternative approach defines icephobicity as the ability to prevent ice formation on the surface^17,18^. Icephobic should be, therefore, a surface with a minimum ice nucleation temperature. Such surfaces can be successfully operated even at a few degrees above this temperature, allowing an extended freezing delay time^18^. The optimal texture developed on such surface should have multitier structuring, designed also to account for heterogeneous nucleation thermodynamics, to simultaneously reduce and optimize the liquid–solid contact area (lower nucleation temperature) and to improve the droplet mobility, namely to reduce contact time, and to prevent the Cassie–Wenzel wetting transition^19^. And it was concluded that such surfaces should be designed for the specific environmental conditions^18^. Obviously, design of the tailor-made surfaces preventing ice formation calls for profound understanding of water nucleation on the micro- and nano-rough interfaces^20,21^. It should be emphasized that superhydrophobic surfaces, with very high receding contact angle may have strong adhesion to ice^22^. It was demonstrated recently that there exists the critical confinement length scale enabling catching of liquid water (as opposed to ice) in between roughness features and thus reducing the strength of ice adhesion by over a factor of twenty-seven compared to traditional hydrophobic surfaces^17^. The reduction in ice adhesion is due to the meta-stability of liquid water; as ambient ice cleaves from the textured surface, liquid water leaves confinement and freezes—a process which takes the system from a local energy minimum to a global energy minimum^17^. This phase transition generates a detachment force that actively propels ambient ice from the surface^17^. Finally, the third, alternative approach to the design of icephobic interfaces was suggested, implying bouncing of incoming small droplets at the temperatures below the freezing point^23–25^. Generally, icephobic surfaces should: prevent freezing of water condensing on the surface, prevent freezing of incoming water if ice formed, and they should have as weak adhesion strength with the solid as possible^1,3,12,25^. In spite of a much effort invested into the development and manufacturing of icephobic surfaces the interfaces, which repel efficiently ice, remain scarce^24–27^. We conclude that the development of the effective anti-icing surfaces remains a challenging scientific and engineering problem. We report the robust ice-repelling surface based on the urchin-like Al_2_O_3_ particles.

## Experimental: materials and methods

### Materials

**Figure 1:**
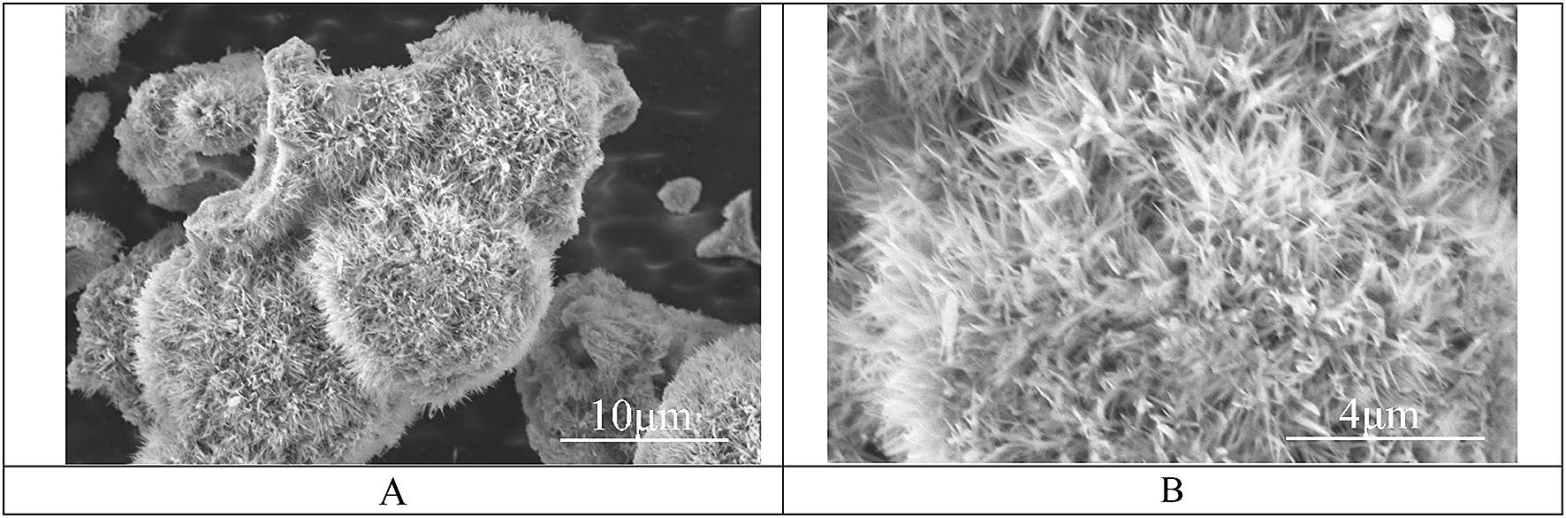
SEM images of the urchin-like Al_2_O_3_ particles, synthesized according to the protocol, supplied in Ref.^28^. (**A**) Scale bar is 10 μm. (**B**) Scale bar is 4 μm.

Epoxy oligomer (MW = 400, Tetraethyl orthosilicate (TEOS), 1H,1H,2H,2H-perfluorodecyltrichlorosilane (FDTS)) and 3-aminopropyltriethoxysilane (abbreviated APTES) were supplied by Alfa Aesar, GB. Aluminum sulfate, Aluminum nitrate, Dimethyl sulfoxide ≥ 99.5% (DMSO), Ethyl Alcohol 96.0–97.2%, were supplied by Sigma-Aldrich, 2-propanol 98%, Urea 99% were supplied by Acros Organics. Urchin-like spherule particles of Al_2_O_3_ were synthesized by the hydrothermal method described in detail in Ref.^28^. SEM images of the particles are depicted in Fig. 1. The average diameter of the spherules was $10\pm 1$ μm.

### Methods

#### Manufacturing of “urchin”-like particles of Al_2_O_3_

Urchin-like particles were obtained by the method of hydrothermal synthesis, from an aqueous-alcoholic solution of aluminum salts taken at a ratio of Al_2_(SO_4_)_3_:Al(NO_3_)_3_ = 80:20 ml%/mol%. The molar ratios of the components of the synthesis were Al^3+^/CO(NH_2_)_2_/H_2_O/i-PrOH = 1:2:100:5, the synthesis scheme is described in detail in^28^.

Particles were hydrophobized as follows: at the first stage of the process 0.4 g of FDTS were introduced into 50 ml of hexane. 4.0 g particles of Al_2_O_3_ were introduced into the solution under mixing concentration 7.5%. The suspension was homogenized with the ultrasonic disperser Bandelin Sonopuls HD 3200 (modulus KE 76) during 2 min. After this Al_2_O_3_ particles were dried under $t=50$ °C during 3 h. The manufactured hydrophobized urchin-like Al_2_O_3_ particles were used for manufacturing of the icephobic, omniphobic coating.

#### Manufacturing of the icephobic, omniphobic coating

**Figure 2:**
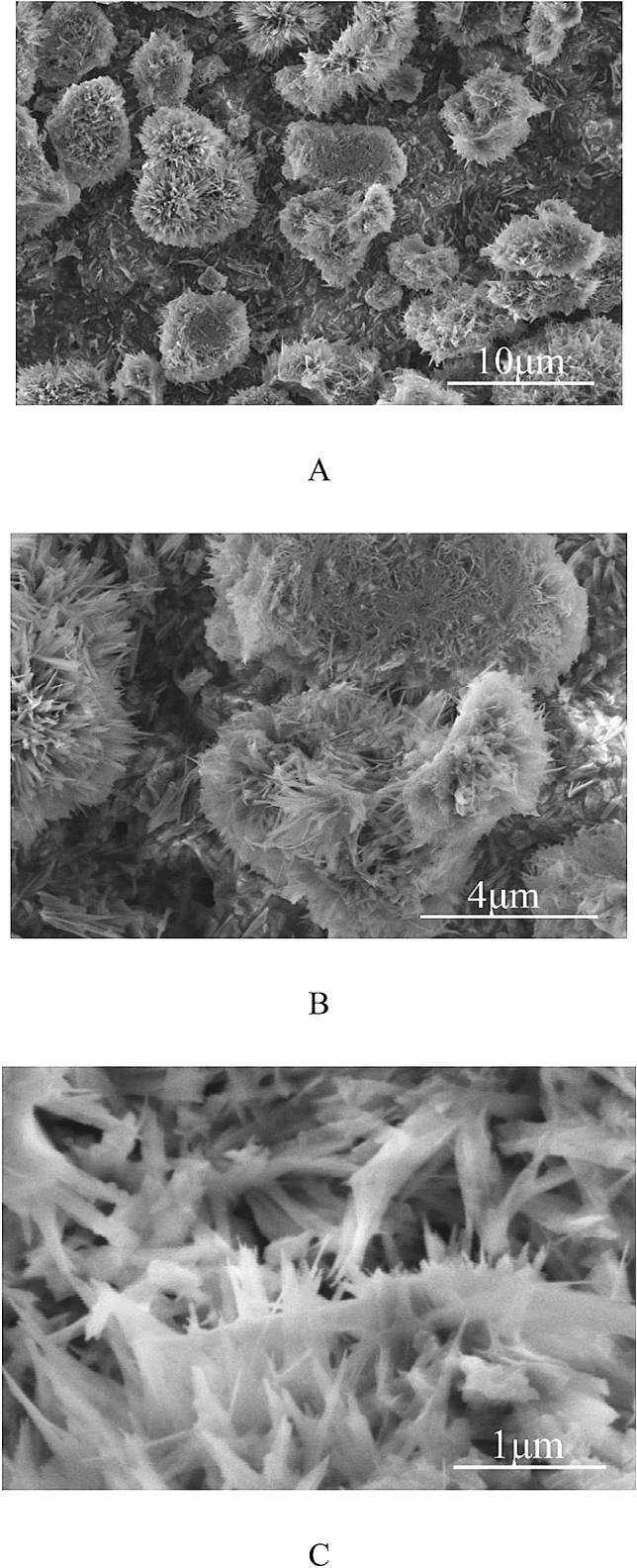
SEM image of the omniphobic, icephobic surface is supplied. (**A**) Scale bar is 10 μm. (**B**) Scale bar is 4 μm. (**C**) Scale bar is 1 μm. The very similar topographies were registered for the surfaces obtained with the dip—coating and spraying methods.

The icephobic coating was manufactured according to the following protocol: 1 g of APTES was mixed with 1.5 g of TEOS and 0.3 g of the epoxy oligomer. Mixing was performed with the magnetic stirrer during 10 min. In the course of the mixing 0.4 g of Al_2_O_3_ were introduced and homogenized with ultrasound during 30 s. The total energy necessary for homogenization was estimated as 0.8 kJ. The obtained suspension was deposited on the solid substrate by spraying or dip coating. Al and Ti plates with the thickness of 0.1 and 1 mm, cleansed by ultrasound, ethanol and acetone, were used as the solid substrates. Solid substrates coated with the omniphobic coating were exposed to the thermal annealing under the temperature of 150 °C during 1 h. Thermal annealing was followed by dipping of the solidified coating into 0.1% wt. hexane solution of FDTS. The coating was dried under 20 °C during 5 min, and finally annealed under the temperature of 150 °C during 1 h. SEM images of the eventual coating are supplied in Fig. 2.

#### Study of the topography, morphology and the chemical composition of the coating

**Figure 3:**
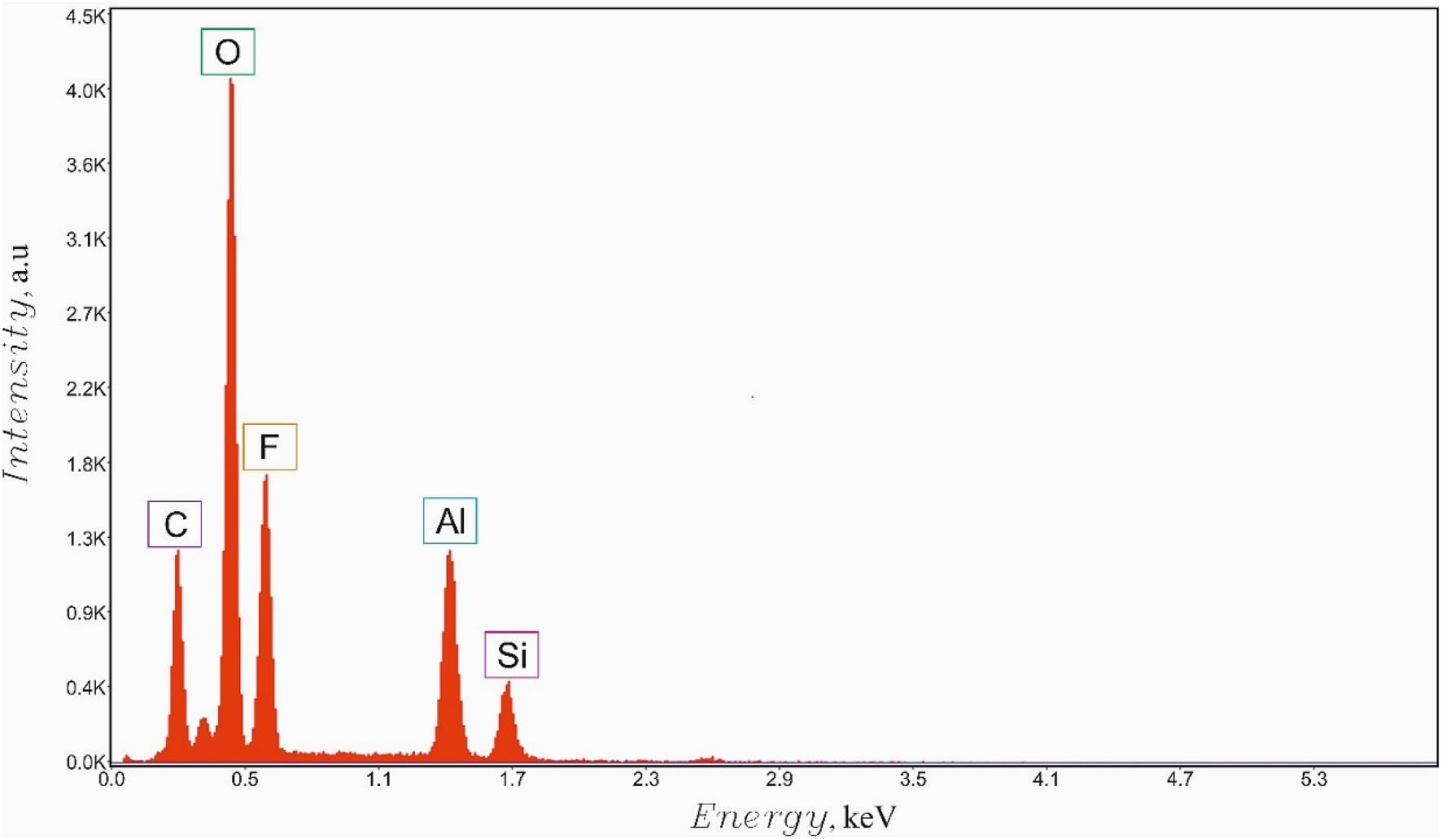
EDS analysis of the icephobic, omniphobic surface is supplied. The pronounced fluorination of the surface is recognized.

Topography and morphology of the urchin-like Al_2_O_3_ particles manufactured as discussed in “Manufacturing of “urchin”-like particles of Al_2_O_3_” and the eventual omniphobic coating, prepared as reported in “Manufacturing of the icephobic, omniphobic coating”, were studied with scanning electron microscope with auto-emission cathode (FE-SEM, FEI, Quanta 650FEG). Chemical composition of the samples was investigated with the energy dispersive X-ray spectrometry (EDS, EDAX Octane Elite). Typical results of the EDS spectroscopy of the reported surfaces are illustrated with Fig. 3.

Wetting characteristics of the coating (apparent contact angles and the contact angle hysteresis) were measured with the precise goniometric system (Kruss DSA-100). 8 $\upmu {\text{l}}$ de-ionized water droplets were used for the study of the wetting characteristics of the reported icephobic, omniphobic surfaces. The results were averaged across 10 measurements.

Contact angle hysteresis was established with the needle-syringe method. 5 μl droplet was placed on the surface and inflated with a syringe; the triple (contact) line was pinned to the substrate up to a certain volume of the droplet. When the triple line was pinned, the contact angle increased till a certain threshold value *θ*_A_ beyond which the triple line started to move. The threshold contact angle *θ*_A_ was registered as the advancing contact angle^10^. When a droplet was deflated with a syringe its volume was decreased to a certain limiting value; in parallel the contact angle decreases till a threshold value *θ*_R_, registered as the receding contact angle, denoted ${\theta }_{R}$. When *θ* = *θ*_R_, the triple line suddenly moved. Both *θ*_A_ and *θ*_R_ are equilibrium, although metastable contact angles^10^. The difference between *θ*_A_ and *θ*_R_, namely $\Delta \theta ={\theta }_{A}-{\theta }_{R}$ is known as the contact angle hysteresis^10^.

In parallel, sliding angles were established with the inclined (tilted) plane method. The minimal angle at which 5–10 µl water droplets started to slide along the studied surface was registered. The establishment of the contact angle hysteresis and the sliding angle were performed at the ambient conditions ($t=25\;^\circ\mathrm{C}).$

#### Study of the anti-icing properties of the surfaces

**Figure 4:**
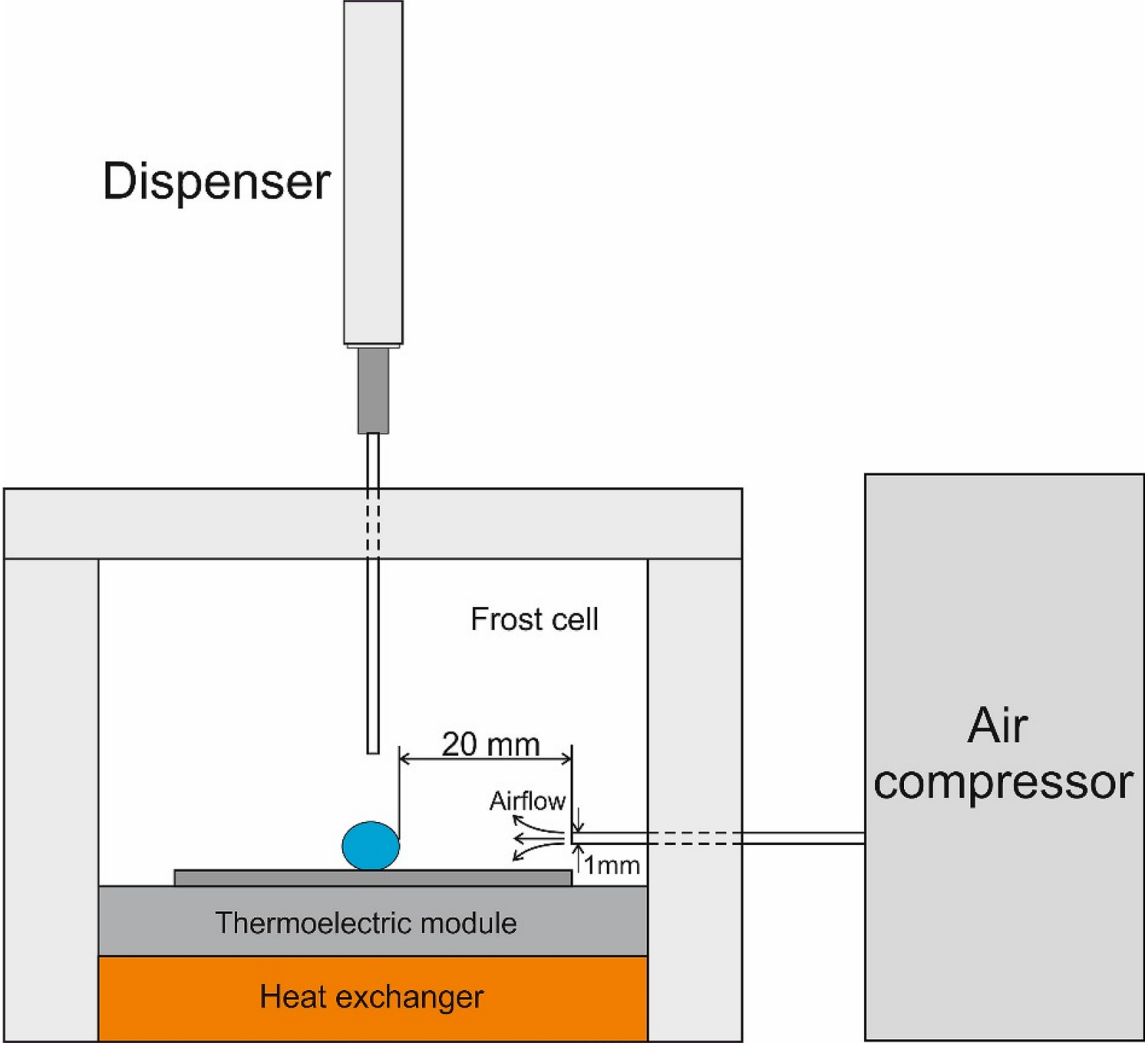
Scheme of the experimental unit used for testing of the anti-icing properties of the reported surfaces is depicted.

2 μl droplet of de-ionized water was placed on the cooled omniphobic surface, manufactured as described in “Manufacturing of “urchin”-like particles of Al_2_O_3_” and “Manufacturing of the icephobic, omniphobic coating”. Apparent contact angle (abbreviated APCA) was measured immediately after dripping of the droplet and in the course of cooling of the surface. Cooling from $t=20$ to − 15 ± 0.5 °C was performed with the thermo-electric modulus with a power of *W* = 95 W, enabling the maximal temperature change of 84 °C. The cooling rate was 5 ℃/min. Cooling was performed with thermo-electric modulus under circulation of refrigerant trough the copper heat exchanger. The surface temperature was controlled with the dual laser IR sensor and the *K*-type thermocouple. The omniphobic surface was cooled from ambient conditions to *t *= − 15 ± 0.5 °C and the apparent contact angle was measured every 15 s; apparent contact angle was taken also in a course of crystallization. Initial temperature and humidity in the experimental cell were set as *t* = 25 °C; $RH=40\pm 1\%.$ Humidity was registered with the humidity meter CEM DT-625. Relative humidity was increased in the course of cooling to *t* = − 15 ± 0.5 °C and was stabilized at the level of $RH=60\pm 1\%.$ Then the cooling was disconnected and the melting of ice crystals was observed; the apparent contact angle was taken in a course of melting. Water droplets were removed from the surface with the air jet (velocity $v=3.0\pm 1.0$ m/s). The testing was carried out with the experimental cell described in Fig. 4; t repetitions of testing procedures were performed. Air stream was controlled with the compressor, supplying compressed air with the volumetric flow rate of $\dot{V}=1-9\frac{\mathrm{ml}}{\mathrm{s}}$; air consumption at $v=2.0\frac{\mathrm{m}}{\mathrm{s}}$ was $\dot{V}=1.6\pm 0.1\frac{\mathrm{ml}}{\mathrm{s}}$. Geometrical parameters of the cell are supplied in Fig. 4. The diameter of the air outlet was 1 mm.

## Results and discussion

### Wetting properties of the surfaces comprising urchin-like Al_2_O_3_ particles

**Figure 5:**
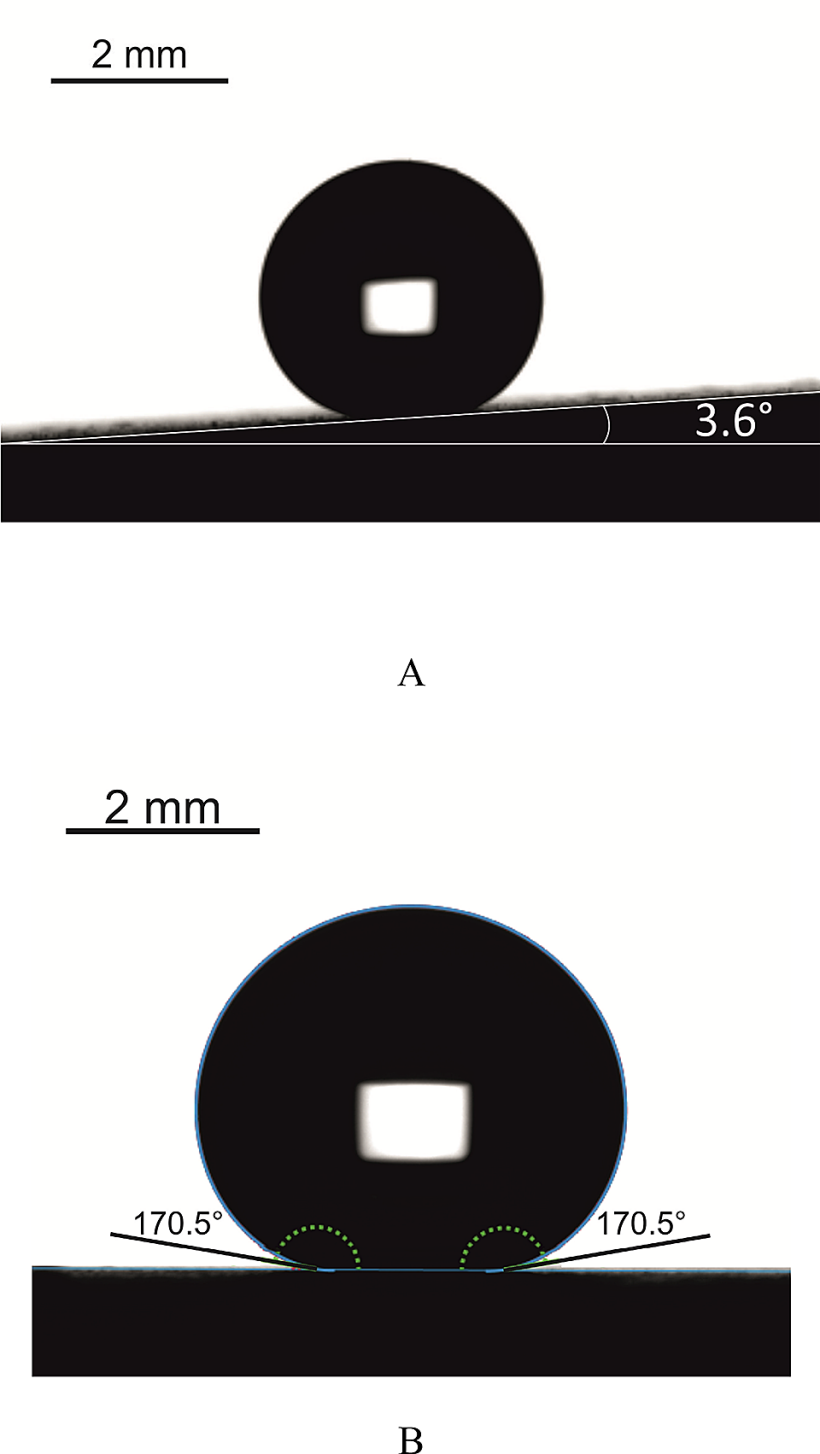
(**A**) Sliding angle of 3.6° established for 5 μl water droplets deposited on the omniphobic surfaces is shown. (**B**) Apparent contact angle $\theta =170.{5\pm 1}^{\circ}$ is shown.

Address first the topography of interfaces, manufactured as described in the “Experimental” section and illustrated with Fig. 2. The reported surface demonstrates two distinct spatial scales, namely the “large scale” (“urchins”) which is *ca* 10 μm and the small scale (“needles”) which is 10 nm. The needles are fluorinated as evidenced by the EDS spectroscopy, illustrated with Fig. 3. Fluorination of the interface did not change its topography, as shown in Supplementary Image Fig. S1. Fluorinated surfaces comprising urchin-like Al_2_O_3_ particles demonstrated pronounced omniphobic properties, namely APCA values as high as $\theta =170.5\pm 1^\circ$ (see Fig. 5) and the low contact angle hysteresis were registered for these surfaces, namely $\Delta \theta ={\theta }_{A}-{\theta }_{R}=6.5\pm 0.5^\circ$. Spraying and dip coating methods used for manufacturing of the reported surfaces supplied very close topographies and wetting and anti-icing properties, and, thus, considered below together. The radius of 5 μl droplet was close to $R\cong 1$ mm and it was smaller than the water capillary length ${l}_{ca}\cong 2.74$ mm; thus, water droplets kept shape which was close to spherical, as depicted in Fig. 5.

**Figure 6:**
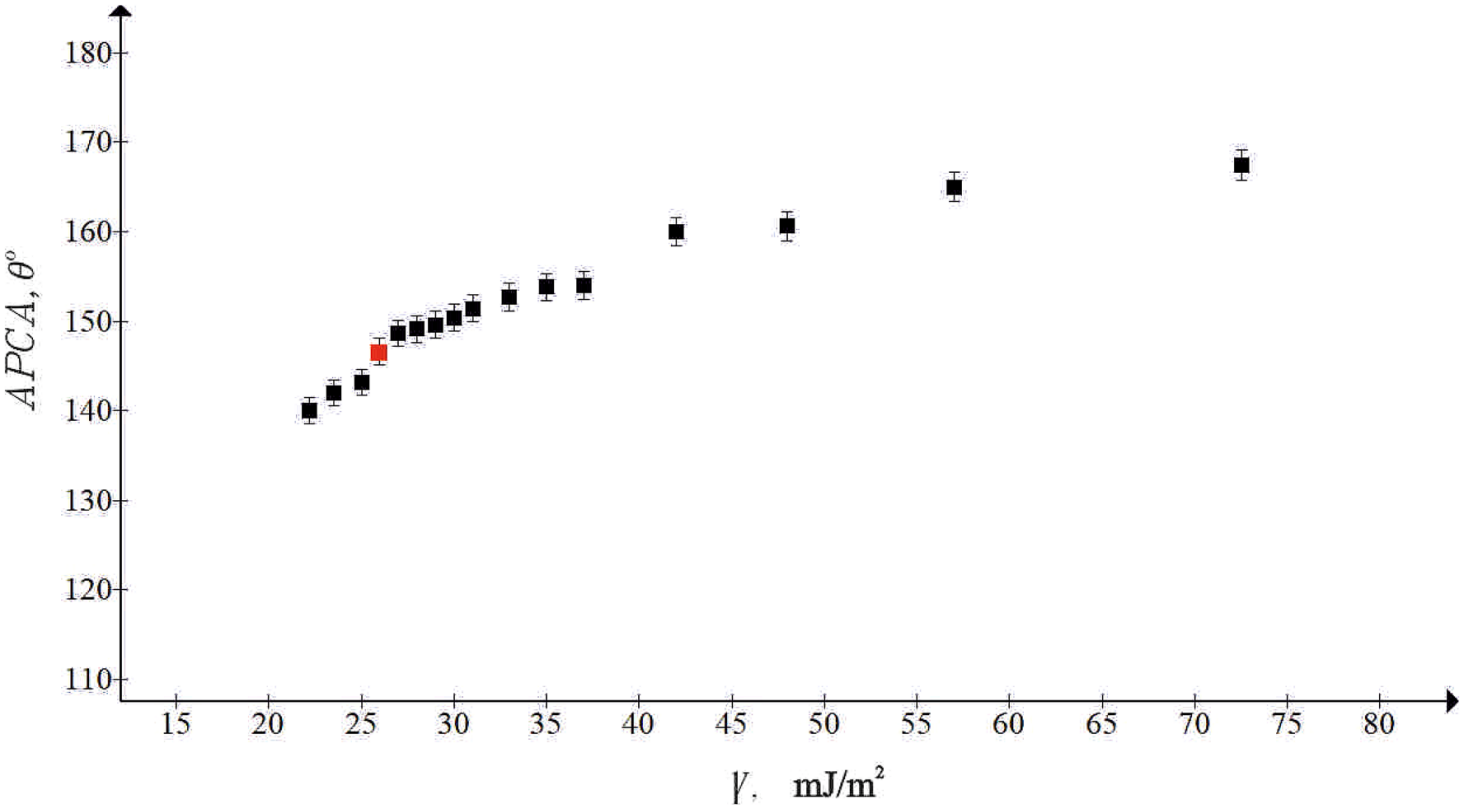
The experimentally established dependence of the apparent contact angle vs. the surface tension of aqueous ethanol solution is presented. Red point depicts the Cassie–Wenzel wetting transition.

Sliding angles as low as 3.6° established for 5 μl water droplets were registered as depicted in Fig. 5. High apparent contact angles and low contact angle hysteresis themselves evidence but do not guarantee the true superhydrophobicity and omniphobicity of the surface; the high stability of the Cassie wetting state is also necessary^19,29–35^. Various experimental techniques were implemented for the study of the stability of the Cassie wetting state^19^. We tested the stability of the Cassie air trapping wetting with the technique enabling establishment of the threshold (critical) value of the surface tension of liquid (denoted ${\gamma }_{c} )$ at which onset of the Cassie–Wenzel transition is registered, which was used successfully by a number of research groups for this purpose^36–38^. Droplets of aqueous ethanol solutions were placed on the reported surface, and the apparent contact angle was taken. Figure 6 depicts the dependence of the APCA taken on the studied surfaces on the surface tension of the tested aqueous alcohol solutions. The concentration of ethanol in droplets was gradually increased, and consequently the apparent contact angle decreased. Decrease in the APCA is not abrupt, but it is continuous, and we assume that the onset of the Cassie–Wenzel wetting transitions corresponds to the wetting regime at which APCA becomes smaller that 150°^36–38^.

The red point in Fig. 6 indicates the onset of the wetting transition^36–38^. It is recognized from Fig. 6 that the onset of the Cassie–Wenzel transition corresponds to the ${\gamma }_{c}\cong 30-35\frac{\mathrm{mJ}}{{\mathrm{m}}^{2}}$.

**Figure 7:**
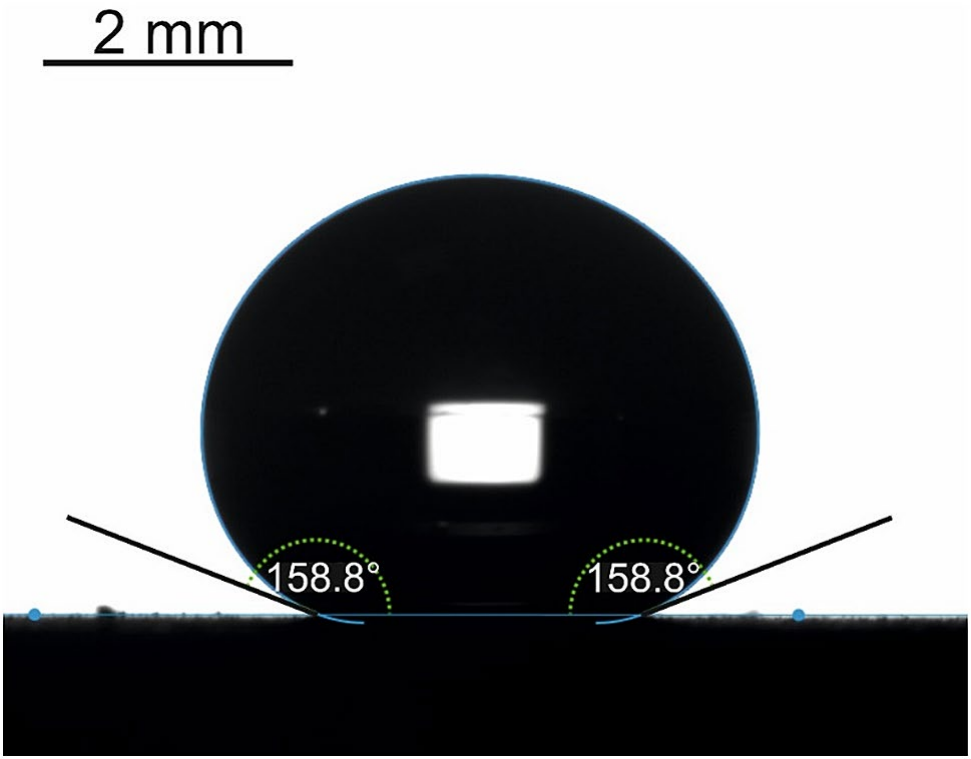
5 μl DMSO droplet placed on the “urchin”-like fluorinated surface is shown. Apparent contact angle is $\theta =158.{8\pm 1}^{\circ}$, the sliding angle was 15°.

This finding evidences the pronounced omniphobicity of the reported surfaces, displayed in Fig. 2. Omniphobicity of the reported surfaces is proved by high APCA of 8 μl dimethyl sulfoxide (DMSO) droplets (the surface tension at ambient conditions $\gamma \cong 43.54\frac{\mathrm{mJ}}{{\mathrm{m}}^{2}}$^39^), which are $\theta =159\pm {1}^{\mathrm{o}}$, as shown in Fig. 7.

The surface tension of DMSO $\gamma >{\gamma }_{c}$ and DMSO droplets, indeed, demonstrated high APCA accompanied with low sliding angles of 15 ± 1°; thus, evidencing the Cassie air trapping wetting regime. It should be emphasized, that urchin-like non-fluorinated surfaces demonstrated complete wetting by water (in other words, APCA was zero). It is reasonable to suggest that the omniphobicity of the reported surfaces emerges from an interplay of the hierarchical relief^40,41^ with its fluorination, illustrated with Fig. 3. Fluorination decreases the specific surface energy of the surface, thus strengthening its omniphobicity^42,43^. It is noteworthy, that the established critical value of surface tension was markedly smaller than that reported in Ref.^43^, namely ${\gamma }_{c}\cong 40\frac{\mathrm{mJ}}{{\mathrm{m}}^{2}}$; thus, underlining pronounced omniphobicity of the reported urchin-like surfaces.

### Anti-icing properties of the surfaces comprising urchin-like Al_2_O_3_ particles

It should be emphasized that hydrophobic and even omniphobic properties of the interface do not ensure its icephobicity, as discussed in Refs.^2,3,22^. Highly developed superhydrophobic surfaces sometimes promote heterogeneous nucleation of ice, as mentioned in Refs.^20,21^. Contrastingly, the reported surfaces demonstrate the pronounced icephobic properties. Consider first water crystallization, which took place on the cooled flat aluminum surfaces. Crystallization was accompanied with the change in the transparency of droplets: this change, evidencing formation of ice was registered at $t=10$ s of cooling. Crystallization of water droplets on the flat surfaces at the cooling rate of 5 ℃/min started at *t* = − 7 ℃, and it finally was registered after 90 s of cooling under the APCA $\theta ={40\pm 1.0}^{\mathrm{o}}$. Water droplet placed on the surface built on the non-fluorinated urchin-like Al_2_O_3_ particles was frozen after 3 min of cooling. It was impossible to remove droplets from the surface with an air jet.

**Figure 8:**
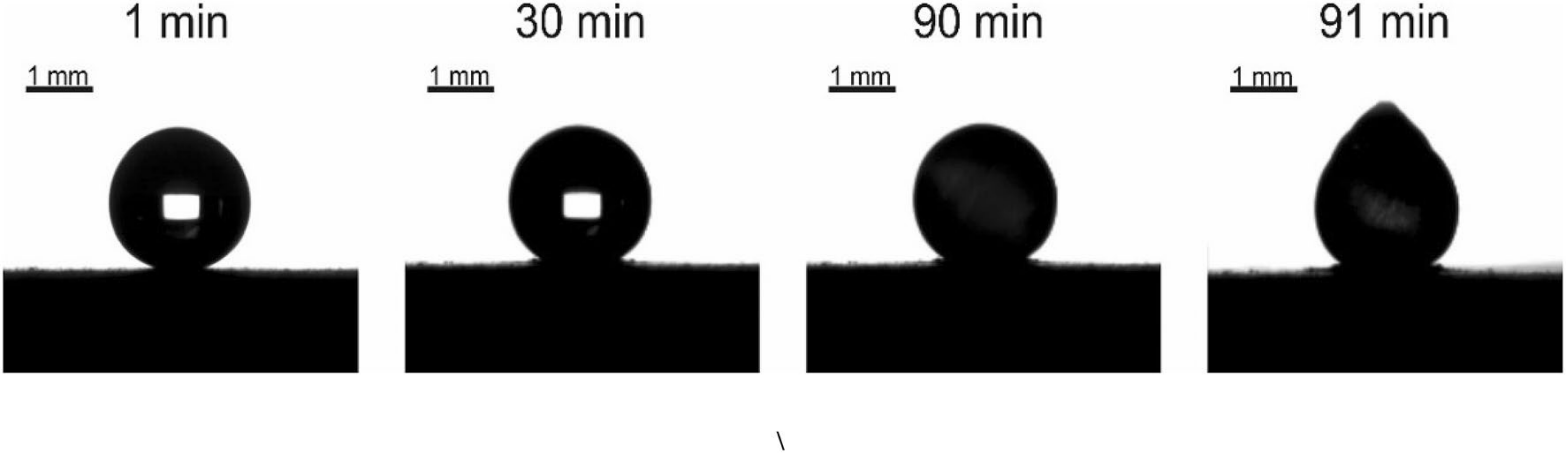
Time evolution of the shape of the 2 μl water droplet placed on the cooled icephobic surface at *t* = − 15 °C at $RH=80\%$ is shown. The typical tip is formed at the top of the droplet at final stage of cooling. Scale bar is 1 mm.

**Figure 9:**
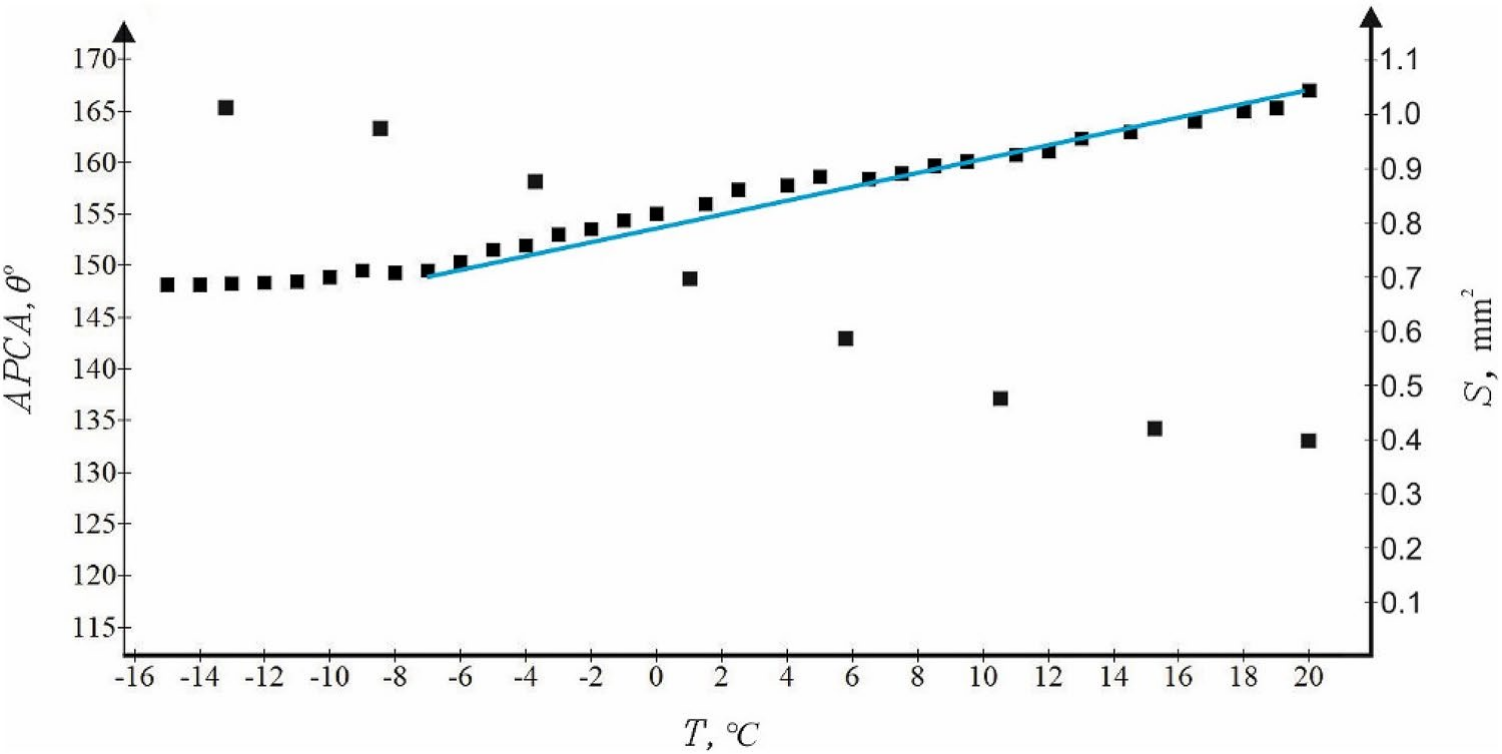
Dependences of APCA $\left(\theta \right)$ and the contact area (*S*) on the temperature of the supporting surface are depicted. The blue straight line illustrates the linear approximation of the temperature dependence of APCA in the range − 6 °C < *t* < 22 °C; $\frac{d\theta }{dT}\cong {10}^{-2}\frac{rad}{K}$ . The contact area *S* is stabilized at *t* ≅ − 10 °C, thus evidencing completion of ice crystallization at the contact area.

Now address water crystallization observed on the fluorinated “urchin”-like surfaces and illustrated with the Fig. 8 and Supplementary Movie 1. Consider first the dependence of APCA on the temperature of the supporting fluorinated urchin-like icephobic surface, shown in Fig. 9. It is recognized from the data supplied in Fig. 9 that APCA decreased in a course of cooling during $1\pm 0.1$ min from ambient conditions to *t* = − 6 ℃. The rate of this decrease is approximately $\frac{d\theta }{dT}\cong {10}^{-2}\frac{\mathrm{rad}}{\mathrm{K}}$. It should be emphasized that at *t* = − 6 ℃ the APCA was stabilized at the high level of $\theta =148\pm {1}^{\circ}$. It is reasonable to suggest that at the aforementioned temperature interface started to crystallize, and thus, it was transformed to the solid–solid (namely, ice/fluorinated urchin-like surface) interface. Thus, we observed the so-called “interfacial water crystallization”, which was deeply studied by investigators^20,21,43,44^.

**Figure 10:**
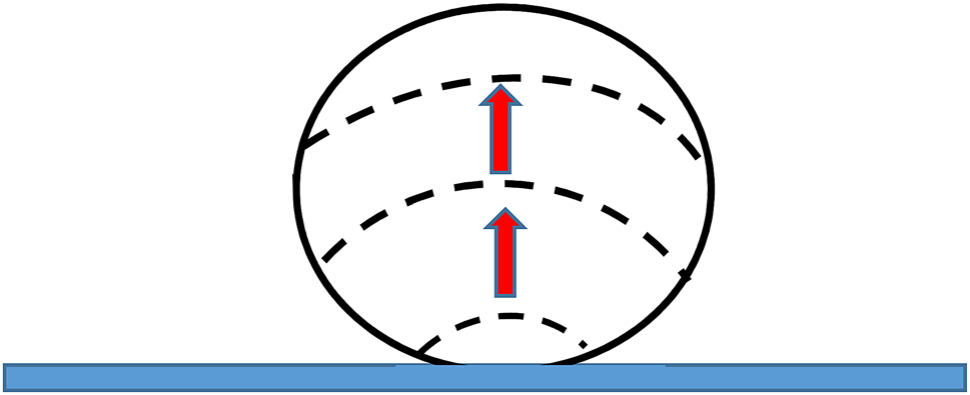
Scheme of displacement of the crystallization front (shown with a dashed line) within a droplet cooled on the omniphobic surface decorated with the urchin-like Al_2_O_3_ particles is shown. Red arrow demonstrates the direction of the displacement of the crystallization front, the characteristic time of the displacement of the crystallization front within 5–10 μl droplet is estimated as 10–30 s.

Interfacial crystallization was followed by the bulk crystallization, which started with an essential delay to be addressed below in detail. Bulk crystallization of water within a cooled droplet was accompanied with the displacement of the crystallization front. The propagation of the crystallization front within the droplet, which started from the contact area, is illustrated with the Supplementary Video 1 and Fig. 10. Water turbidity within a droplet was registered before the onset of the propagation of the crystallization front, with the temporal separation of $\widehat{\tau }\cong 1-2$ s. Perhaps, this time span may be interpreted as the nucleation time scale.

The characteristic time of the movement of the front of crystallization within a droplet, depicted schematically with Fig. 10, was established as 10–30 s for the 5–10 μl droplets. Thus, the velocity of propagation of the crystallization front is established as ${v}_{fr}\cong 0.15\pm 0.05\frac{\mathrm{mm}}{\mathrm{s}}$. Establishment of ${v}_{fr}$ enabled calculation of the thermal Peclet number according to Eq. (1):

1 $$Pe=\frac{R{v}_{fr}}{\alpha },$$

where *R* is the radius of the droplet and *α* is the thermal diffusivity of water at *t* = 0 °C$;$ assuming $R\cong 1 \mathrm{mm} \mathrm{and} \alpha \cong {1.0\times 10}^{-7}\frac{{\mathrm{m}}^{2}}{\mathrm{s}}$ (see Ref.^45^) yields $Pe\cong 1.5$. This estimation means that the thermal advection within a droplet is comparable to thermal diffusivity and it implies that the rigorous quantitative analysis of the heat transfer within the cooled, crystallized droplet is well expected to be perplexed.

At the temperature* t* = − 6 ± 0.5 °C, the rate of crystallization was markedly slowed; cooling procedure which started at *t* = − 6 ± 0.5 °C and continued for $2\pm 0.1\;{\text{min}}$ was stopped at *t* = − 15 ± 0.5 °C. Water within a droplet was completely crystallized after ${\tau }_{cr}=85\pm 5$ min of isothermal cooling at the temperature of *t* = − 15 ± 0.5 °C. Thus, the pronounced total time delay of the bulk ice crystallization (i.e. freezing delay time span) as high as ${\tau }_{del}\cong 88\pm 5\;{\text{min}}$ was registered (as fixed from the onset of the cooling from ambient conditions). Equation (1) and experimental observations enabled development of the following hierarchy of the time scales:

2 $${\tau }_{cr}\gg {\tau }_{therm}\cong {\tau }_{prop}\gg \widehat{\tau },$$

where ${\tau }_{therm}\cong \frac{{R}^{2}}{\alpha }\sim 10$ s is the time scale of the thermal equilibration within a cooled droplet and ${\tau }_{prop}\cong \frac{2R}{{v}_{fr}}\sim 10-30$ s is the time scale of propagation of the crystallization front. In other words, the characteristic time of nucleation is much smaller than the comparable time scales ${\tau }_{therm}$ and ${\tau }_{prop}$ and propagation of the crystallization front within a droplet occurs, when a droplet is far from its thermal equilibrium. The time scale corresponding to the onset of the bulk crystallization ${\tau }_{cr}$ is much larger than all of the aforementioned time scales.

The delay of the bulk ice crystallization was markedly larger than the maximal time span of the delay reported in Ref.^46^, namely ${\tau }_{del}\cong 12\;{\text{min}}$. It is noteworthy that ice crystallization started from the solid/liquid interface; thus, evidencing its interfacial nature. It should be emphasized, that after thawing of the surfaces the apparent contact angle of water droplets restored its initial high value, evidencing the superhydrophobicity of the surface. Thawed droplets were easily removed by the low speed air jet. This observation enables multiple, cyclic use of the suggested icephobic surfaces.

The delay in crystallization is reasonably related to the temperature increase within the droplet due to the release of latent heat of fusion, as suggested in Ref.^46^. The high values of the APCA under crystallization evidence that the crystallization occurs within the Cassie wetting regime. This assumption is supported by the fact that the crystallized droplets are easily blown out of the surface by the air stream with the velocity $v=3.0\pm 1.0$ m/s, as shown in Supplementary Video 2. This value of velocity is much lower than that inherent for aircraft and turbine applications of the icephobic surfaces. The characteristic time scale of taking away of a droplet of the surface by the air jet was established as $\tau \cong 2.5\pm 0.5$ s.

Crystallization was accompanied with the change in the area of contact area and the shape of the cooled droplet, as shown in Fig. 8. The characteristic tip inherent for crystallized droplets appeared at the eventual stage of cooling, as shown in Fig. 8. The contact area grew in a course of cooling, as shown in Fig. 9. Such a behavior is typical for droplets cooled on superhydrophobic surfaces, as discussed in Ref.^44^. The reasonable explanation of this effect was proposed in Ref.^46^; namely, it was suggested that the supersaturation of water vapor in the proximity of the triple line influences the solid–vapor and solid–liquid surface tensions. Consequently, the surface appears more hydrophilic at decreasing surface temperatures. The increase in contact area between the cooled droplet and the surrounding vapor occurs despite the increase in the surface tension of water in a course of cooling^46,47^. The contact area was stabilized within the range of temperatures of ∆*t* = − 6 – (– 10) °C, as shown in Fig. 9; thus, evidencing completion of ice crystallization at the contact area. We conclude that the urchin-like, twin-scale fluorinated Al_2_O_3_ surfaces demonstrate the pronounced icephobic properties when compared with flat and non-fluorinated ones. Crystallized ice is easily removed from the surfaces. Heated surfaces restored their omniphobic and icephobic surfaces.

## Conclusions

We report twin-scaled fluorinated surfaces built of “urchin”-like fluorinated Al_2_O_3_ particles. The highly developed hierarchical surface demonstrates two well separated spatial scales, namely the large scale “urchins” which is *ca* $10 \mathrm{\mu m}$ and the nano-scaled “needles”^39,40^.

The surface demonstrated distinct omniphobicity. The onset of the Cassie–Wenzel wetting transitions corresponds to the critical value of liquid ${\gamma }_{c}\cong 30-35\frac{\mathrm{mJ}}{{\mathrm{m}}^{2}}$. In other words, liquids with the larger values of the surface tension are well expected to demonstrate the Cassie air trapping wetting regimes, accompanied by high contact angles and low contact angle hysteresis, when placed on the surface. This assumption was validated by placing of DMSO droplets on the reported surfaces. DMSO demonstrated distinct Cassie-like wetting on the fluorinated “urchin”-like surfaces. Thus, the introduced hierarchical surfaces demonstrate high apparent contact angles, low contact angle hysteresis and high stability of the Cassie wetting state^19,48^.

The reported urchin-like hierarchical fluorinated surfaces also demonstrate pronounced icephobicity. The essential time delay of the bulk ice crystallization as high as *ca* 88 min was registered when compared to the ice formation on the flat aluminum and non-fluorinated “urchin”-like surfaces. Crystallization of ice started from the water/solid interface^20,21,44^; the velocity of propagation of the crystallization front was established experimentally as ${v}_{fr}\cong 0.15\pm 0.05\frac{\mathrm{mm}}{\mathrm{s}}$. The thermal Peclet number was estimated as $Pe\cong 1.5;$ this means that the thermal advection within a droplet is comparable to thermal diffusivity. The hierarchy of the time scales inherent for the thermal processes occurring within a droplet is elucidated. Crystallized ice was easily blown out of the surface by the air stream with the velocity of $v=3.0\pm 1.0\frac{\mathrm{m}}{\mathrm{s}}$, which is much lower than that inherent for exploitation of aircrafts and turbines^1,12^. Heated “urchin”-like surfaces completely restored their omniphobic and icephobic surfaces after thawing, which is extremely important for the industrial applications of the reported surfaces. The pronounced omniphobic and icephobic properties of the hierarchical “urchin”-like surfaces emerge from the combination of the twin-scale topography and fluorination of the interface, decreasing its specific surface energy^1,3,4,12,13^. In our future investigations we plan to study condensation of droplets on the reported “urchin”-like fluorinated surfaces^46^.

## Supplementary Information

Supplementary Figure S1.

Supplementary Video Legends.

Supplementary Video 1.

Supplementary Video 2.

## References

[1] Irajizad P, Nazifi S, Ghasemi H (2019). Icephobic surfaces: Definition and figures of merit. Adv. Colloid Interface Sci..

[2] Jung S, Dorrestijn M, Raps D, Das A, Megaridis CM, Poulikakos D (2011). Are Superhydrophobic surfaces best for icephobicity?. Langmuir.

[3] Hejazi V, Sobolev K, Nosonovsky M (2013). From superhydrophobicity to icephobicity: Forces and interaction analysis. Sci. Rep..

[4] Meuler AJ, McKinley GH, Cohen RE (2010). Exploiting topographical texture to impart icephobicity. ACS Nano.

[5] Ozbay S, Yuceel C, Erbil HY (2015). Improved icephobic properties on surfaces with a hydrophilic lubricating liquid. ACS Appl. Mater. Interfaces.

[6] Stone HA (2012). Ice-phobic surfaces that are wet. ACS Nano.

[7] Golovin K, Kobaku SPR, Lee DH, DiLoreto ET, Mabry JM, Tuteja A (2016). Designing durable icephobic surfaces. Sci. Adv..

[8] Song D, Jiang Y, Chou T, Asawa K, Choi C-H (2020). Spontaneous deicing on cold surfaces. Langmuir.

[9] Sarshar MA, Song D, Swarctz C, Lee J, Choi C-H (2018). Anti-icing or deicing: Icephobicities of superhydrophobic surfaces with hierarchical structures. Langmuir.

[10] Ed. Bormashenko, Physics of Wetting. Phenomena and Applications of Liquids on Surfaces, pp. 20–26, de Gruyter, Berlin, Ge, 2017.

[11] Meuler AJ, Smith JD, Varanasi KK, Mabry JM, McKinley GH, Cohen RE (2010). Relationships between water wettability and ice adhesion. ACS Appl. Mater. Interfaces.

[12] Menini R, Farzaneh M (2011). Advanced icephobic coatings. J. Adhes. Sci. Technol..

[13] Susoff M, Siegmann K, Pfaffenroth C, Hirayama M (2013). Evaluation of icephobic coatings—Screening of different coatings and influence of roughness. Appl. Surf. Sci..

[14] Carreño F, Gude MR, Calvo S, Rodriguez de la Fuente O, Carmona N (2020). Design and development of icephobic coatings based on sol-gel/modified polyurethane paints. Mater. Today Commun..

[15] Irajizad P, Al-Bayati A, Eslami B, Shafquat T, Nazari M, Jafari P, Kashyap V, Masoudi A, Araya D, Ghasemi H (2019). Stress-localized durable icephobic surfaces. Mater. Horiz..

[16] Janjua ZA, Turnbull B, Choy K-L, Pandis C, Liu J, Hou X, Choi K-S (2017). Performance and durability tests of smart icephobic coatings to reduce ice adhesion. Appl. Surf. Sci..

[17] Zhao TY, Jones PR, Patankar NA (2019). Thermodynamics of sustaining liquid water within rough icephobic surfaces to achieve ultra-low ice adhesion. Sci. Rep..

[18] Schutzius TM, Jung S, Maitra T, Eberle P, Antonini C, Stamatopoulos C, Poulikakos D (2015). Physics of icing and rational design of surfaces with extraordinary icephobicity. Langmuir.

[19] Bormashenko E (2015). Progress in understanding wetting transitions on rough surfaces. Adv. Colloid Interface Sci..

[20] Abyzov AS, Schmelzer JWP (2013). Generalized Gibbs’ approach in heterogeneous nucleation. J. Chem. Phys..

[21] Abyzov AS, Davydov LN, Schmelzer JWP (2019). Heterogeneous nucleation in solutions on rough solid surfaces: Generalized Gibbs approach. Entropy.

[22] Nosonovsky M, Hejazi V (2012). Why superhydrophobic surfaces are not always icephobic. ACS Nano.

[23] Liu J, Janjua ZA, Roe M, Xu F, Turnbull B, Choi K-S, Hou X (2016). Super-hydrophobic/icephobic coatings based on silica nanoparticles modified by self-assembled monolayers. Nanomaterials.

[24] Ramachandran R, Sobolev K, Nosonovsky M (2015). Dynamics of droplet impact on hydrophobic/icephobic concrete with the potential for superhydrophobicity. Langmuir.

[25] Zhang S, Huang J, Cheng Y, Yang H, Chen Z, Lai Y (2017). Bioinspired surfaces with superwettability for anti-icing and ice-phobic application: Concept, mechanism, and design. Small.

[26] Coady MJ, Wood M, Wallace GQ, Nielsen KE, Kietzig A-M, Lagugné-Labarthet F, Ragogna PJ (2018). Icephobic Behavior of UV-cured polymer networks incorporated into slippery lubricant-infused porous surfaces: Improving SLIPS durability. ACS Appl. Mater. Interfaces.

[27] Coady MJ, Getangama NNK, Khalili A, Wood M, Nielsen KE, de Bruyn JR, Hutter JL, Klassen RJ, Kietzig A-M, Ragogna PJ (2020). Highly cross-linked UV-cured siloxane copolymer networks as icephobic coatings. J. Polymer Science.

[28] Lebedeva II, Starostin AS, Valtsifer IV, Valtsifer VA (2018). Hydrothermal synthesis of urchin-like alumina for fire-extinguishing powders. J. Mater. Sci..

[29] Nosonovsky M, Bhushan B (2009). Superhydrophobic surfaces and emerging applications: Non-adhesion, energy, green engineering. Curr. Opin. Colloid Interface Sci..

[30] Nosonovsky M, Bhushan B (2008). Patterned nonadhesive surfaces: Superhydrophobicity and wetting regime transitions. Langmuir.

[31] Sahoo BN, Nanda S, Kozinski JA, Mitra SK (2017). PDMS/camphor soot composite coating: Towards a self-healing and a self-cleaning superhydrophobic surface. RSC Adv..

[32] Li Y, Quéré D, Lv C, Zheng Q (2017). Monostable superrepellent materials. PNAS.

[33] Papadopoulos P, Mammen L, Deng X, Vollmer D, Butt H-J (2013). How superhydrophobicity breaks down. PNAS.

[34] Whyman G, Bormashenko E (2012). Wetting transitions on rough substrates: General considerations. J. Adhes. Sci. Technol..

[35] Liu J, Re X, Zhou X (2012). A new look on wetting models: Continuum analysis. Sci. China.

[36] Boreyko JB, Baker CH, Poley CR, Chen C-H (2011). Wetting and dewetting transitions on hierarchical superhydrophobic surfaces. Langmuir.

[37] Whitby CP, Bian X, Sedev R (2013). Rolling, penetration and evaporation of alcohol−water drops on coarse and fine hydrophobic powders. Colloids Surf. A.

[38] Grynyov R, Bormashenko E, Whyman G, Bormashenko Y, Musin A, Pogreb R, Starostin A, Valtsifer V, Strelnikov V, Schechter A, Kolagatla S (2016). Superoleophobic surfaces obtained via hierarchical metallic meshes. Langmuir.

[39] Clever HL, Snead CC (1963). Thermodynamics of liquid surfaces: The surface tension of dimethyl sulfoxide and some dimethyl sulfoxide–acetone mixtures. J. Phys. Chem..

[40] Herminghaus S (2000). Roughness-induced non wetting. Europhys. Lett..

[41] Nosonovsky M, Bhushan B (2007). Hierarchical roughness makes superhydrophobic states stable. Microelectron. Eng..

[42] Yang S, Xia Q, Zhu L, Xue J, Wang Q, Chen Q-M (2011). Research on the icephobic properties of fluoropolymer-based materials. Appl. Surf. Sci..

[43] Bormashenko E, Grynyov R, Chaniel G, Taitelbaum H, Bormashenko Y (2013). Robust technique allowing manufacturing superoleophobic surfaces. Appl. Surf. Sci..

[44] Schmelzer J, Möller J, Gutzow I, Pascov R, Müller R, Pannhorst W (1995). Surface energy and structure effects on surface crystallization. J. Non Cryst. Solids.

[45] James DW (1968). The thermal diffusivity of ice and water between − 40 and + 60 °C. J. Mater. Sci..

[46] Oberlia L, Caruso D, Hall C, Fabretto M, Murphy PJ, Evans D (2014). Condensation and freezing of droplets on superhydrophobic surfaces. Adv. Colloid Interface Sci..

[47] Floriano M, Angell C (1990). Surface tension and molar surface free energy and entropy of water to − 27.2 °C. J. Phys. Chem..

[48] Yao C-W, Tang S, Sebastian D, Tadmor R (2020). Sliding of water droplets on micropillar-structured superhydrophobic surfaces. Appl. Surf. Sci..

