# Supplementary material for: Robust icephobic coating based on the spiky fluorinated Al2O3 particles
Source: Sci Rep. 2021 Mar 8;11:5394. doi: 10.1038/s41598-021-84283-w (PMC7940413; doi:10.1038/s41598-021-84283-w)
Supplement: Supplementary file 1 — Supplementary Figure S1. [file 41598_2021_84283_MOESM1_ESM.pdf]

**Robust Icephobic Coating Based on the Spiky Fluorinated $Al_2O_3$ Particles**

Anton Starostin[a], Vladimir Strelnikov[a], Viktor Valtsifer[a], Irina Lebedeva[a], Irina Legchenkova[b] Edward Bormashenko[b*]

[a]*Institute of Technical Chemistry, UB RAS, Academician Korolev St., 3, 614013 Perm, Russian Federation*

[b]*Engineering Faculty, Chemical Engineering Department, Ariel University, Ariel 407000, POB 3, Israel*

Correspondence to Edward Bormashenko; edward@ariel.ac.il

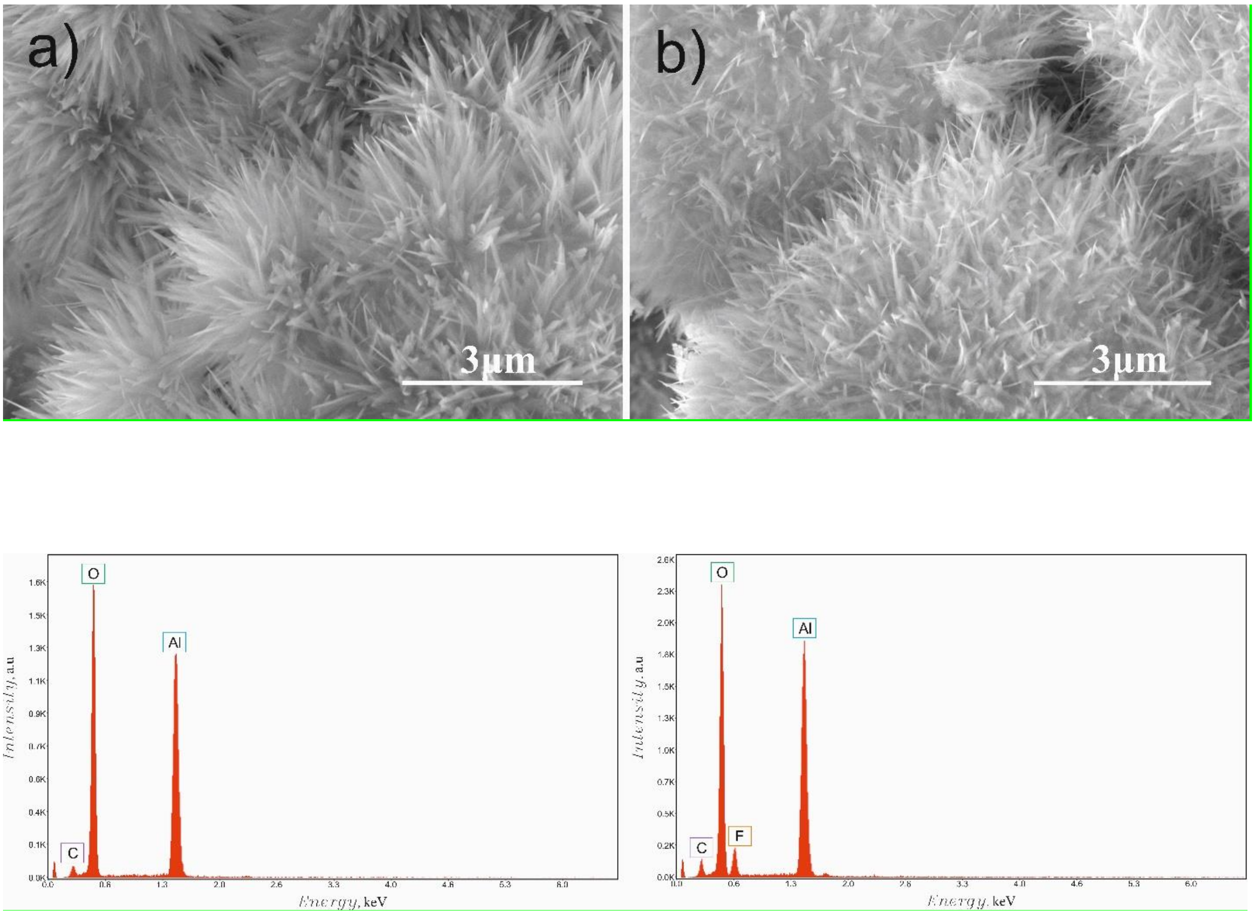


c) d)

**Figure S1**. SEM images of the urchin-like surfaces are presented a) $Al_2O_3$ needles before fluorination b) $Al_2O_3$ needles after fluorination. No changes in the topography is registered.
c) EDS-spectrum of the interface before fluorination; d) EDS-spectrum of the interface before fluorination. Presence of Fluorine is clearly recognized in inset d).
