# Supplementary material for: Robust icephobic coating based on the spiky fluorinated Al2O3 particles
Source: Sci Rep. 2021 Mar 8;11:5394. doi: 10.1038/s41598-021-84283-w (PMC7940413; doi:10.1038/s41598-021-84283-w)
Supplement: Supplementary file 2 — Supplementary Video Legends. [file 41598_2021_84283_MOESM2_ESM.docx]

**Supplementary Materials**

**Supplementary Video 1**. Crystallization of the cooled droplet placed on the fluorinated urchin-like surface is shown. Displacement of the crystallization front within the droplet is clearly seen.

**Supplementary Video 2**. Taking away of the droplet by the air jet from the reported surface is shown.
